# Effect of different blood flow restriction training regimens combined with low-intensity training on muscle strength and cardiovascular safety in older adults: a systematic review and network meta-analysis

**DOI:** 10.3389/fphys.2025.1587876

**Published:** 2025-04-28

**Authors:** Meiling Ren, Guangshen Xian, Xiangchao Tan, Shaocheng Sun, Ming Zhang

**Affiliations:** China Volleyball College, Beijing Sport University, Beijing, China

**Keywords:** blood flow restriction training, low-intensity training, older adults, muscle strength, cardiovascular safety

## Abstract

**Background:**

Older adults are experiencing a gradual decline in physical function as they age. Previous studies have shown that blood flow restriction (BFR) combined with low-intensity training can improve muscle strength and maintain cardiovascular fitness in older adults. However, it remains unclear which training regimen is the most effective. This study aims to investigate the effects of different BFR regimens combined with low-intensity training on muscle strength and cardiovascular safety in older adults.

**Method:**

PubMed, Web of Science, Embase, the Cochrane Library, Scopus, EBSCOhost, and CNKI were searched up to March 2025 to select eligible studies. The randomized controlled trials that explored the effects of BFR training on muscle strength and cardiovascular safety in older adults were included.

**Results:**

A total of 18 studies with 626 participants were included. The results of this network meta-analysis showed that: 1) in terms of improving muscle strength: compared to the controls, low-frequency, low-pressure, and low-intensity BFR training regimen was significantly related to one-repetition maximum (1RM) strength [weighted mean difference (WMD) = 0.58, 95% CI: 0.81–1.08 *P* < 0.05]. Moreover, high-frequency, high-pressure, and low-intensity BFR training was associated with increased muscle cross-sectional area [WMD = 0.50,95% CI (−0.10,1.11), *P* > 0.05] and isometric muscle strength [WMD = 1.44,95% CI (0.75,2.12), *P* < 0.05]; 2) in terms of cardiovascular health: compared to the controls, BFR training regimens at different pressures and frequencies were not linked to changes in heart rate in older adults (*P* > 0.05). Moreover, low-frequency, low-pressure, and low-intensity BFR training regimen was associated with increased systolic blood pressure [WMD = 3.40, 95%CI (0.61,6.19), *P* < 0.05] and diastolic blood pressure [WMD = 13.40, 95%CI (8.96,17.84), *P* < 0.05] in older adults.

**Conclusion:**

Based on the results, high-frequency, high-pressure, and low-intensity BFR training may serve as the optimal regimen to improve muscle strength and maintain cardiovascular fitness in older adults.

**Clinical Trial Registration:** https://www.crd.york.ac.uk/PROSPERO/, **Registration** and protocol CRD42024534387.

## 1 Introduction

In 2024, the World Health Organisation (WHO) stated that the global ageing population continued to rise, with individuals aged 60 and over expected to reach one-sixth of the world’s population by 2030. By 2050, people aged 60 and older will double worldwide (2.1 billion). Ageing mainly impacts the muscular and cardiovascular systems. In terms of the muscular system, ageing leads to a loss of skeletal muscle mass, strength, and fibre size (Grevendonk et al., 2021). Regarding the cardiovascular system, ageing causes a reduction in the ejection fraction and the elasticity of blood vessels (Vakka et al., 2023). The loss of muscle strength and decline in cardiovascular function increase the risk of developing chronic disease in older adults (Zuo et al., 2023). Studies have demonstrated that the prevalence of chronic diseases in older people is significantly higher than in adults, with a prevalence of 81.1 percent in people aged 60 and over (Xiang et al., 2024). Failure to prevent these diseases in older adults will impact their health and cause higher healthcare costs. Training interventions stand as one of the main options to lower the risk of development of these chronic diseases (Vikberg et al., 2019; Zhu et al., 2019). Among the training interventions, traditional resistance training, aerobic exercise (Hughes et al., 2018), and balance exercise (Posadzki et al., 2020) have all been shown to have positive effects on muscle growth. However, these training modalities often require participants to perform high-intensity training with a load value of 70%–80% of 1RM strength for a prolonged period of time (Sharp et al., 2022). Since older adults have lower training tolerance and a higher risk of injury and developing diseases compared to younger adults, their ability to do high-intensity training is limited (Xu et al., 2024). Therefore, the exploration of a training modality that is effective in increasing muscle strength and volume, as well as suitable for the older population is required in medical research.

Blood flow restriction (BFR), also known as KAASTU, is a method of restricting blood flow to proximal muscles by applying a pressure band to the distal limb (Wilkinson et al., 2019). A study has shown that BFR techniques contribute to creating an exercise stimulus. By combining multiple exercise methods, these techniques can enhance body adaptation (Bielitzki et al., 2021). The combination of BFR training with low-intensity muscle contraction exercise (20%–30% of 1RM) (Li et al., 2023) can achieve the effects of high-intensity training (Chua et al., 2022). Therefore, the combination of BFR training and low-intensity exercise can be used as an exercise intervention for older adults to improve their physical functions. BFR training combined with low-intensity exercise enhances muscle hypertrophy and strength gains by elevating metabolic stress (such as lactate accumulation) and hormonal responses (such as growth hormone secretion) (Takarada et al., 2000; Karabulut et al., 2010).

Training programs for older adults need to be carefully designed and managed (Agostini et al., 2023). A study has revealed that BFR pressure levels and exercise frequency are key influencing factors for the effectiveness of muscle growth (Yang et al., 2024). BFR pressure not only affects anabolic and metabolic pathways but also impacts cardiovascular health in older adults. Specifically, appropriate BFR pressure should maximise metabolic stress without completely occluding arterial blood flow or compromising the individual’s safety (McEwen et al., 2019). Furthermore, BFR pressure simultaneously exacerbates the burden on the cardiovascular system. Different levels of pressure have varying effects on physiological functions. Higher pressure during BFR training significantly impacts hemodynamics (Mouser et al., 2019), the autonomic nervous system (Bane et al., 2024), and hormonal responses (Thompson et al., 2024). Moreover, under high-pressure BFR training, metabolic stress is maximized by notably restricted venous return and arterial blood flow, but this may also increase the risk of developing vascular disease (Vervloet et al., 2024). In contrast, low-pressure BFR training partially restricts venous return while preserving arterial blood flow, thus ensuring an adequate supply of oxygen and nutrients during exercise. (Angelopoulos et al., 2021).

Training frequency and duration must be carefully selected to ensure the safety and effectiveness of different pressurised BFR training combined with low-intensity exercise for older adults (Yuan et al., 2023). However, the studies on how these factors influence muscle strength and cardiovascular health in older populations were insufficient and lacked definitive conclusions. Therefore, studies that focus on evaluating the safety and effects of BFR training in older adults, particularly in improving their cardiovascular health, are required. The findings of this study provide effective exercise plans for seniors, thus contributing to improving their training results and reducing health risks. This network meta-analysis compared the effects of different pressures and frequencies of BFR training combined with low-intensity exercise on muscular strength and cardiovascular health in older adults to find the optimal training program, thereby offering insights for clinical decision-making.

## 2 Materials and methods

This study strictly adhered to the guidelines outlined in the Preferred Reporting Items for Systematic Reviews and Meta-Analyses-Network Meta-Analyses (PRISMA-NMA) (Page et al., 2021) and was registered in PROSPERO (CRD42024534387).

### 2.1 Criteria for inclusion and exclusion

The inclusion criteria were as follows: 1) study type: randomized controlled trial and randomized crossover trial; 2) individual age: 55 ≤ age <80 years old (de Jong et al., 2006); 3) intervention: the interventions were categorised as experimental and control groups, with the experimental group performing BFR with low-intensity training (BFR-LI) and the control group (CG) performing low-intensity training; the intensity of the training (low intensity: 20%–30%1RM) (Li et al., 2023); the magnitude of pressurization [high pressure (HP) ≥120 mmHg; low pressure (LP) < 100 mmHg](Mattocks et al., 2019); the frequency [high frequency (HF): ≥3 days/week; low frequency (LF): <3 days/week](Scott et al., 2016); the experimental group used one of the following training regimens: low-frequency, low-pressure, and low-intensity BFR training (the LFLP regimen), low-frequency, high-pressure, and low-intensity BFR training (the LFHP regimen), high-frequency, low-pressure, and low-intensity BFR training (the HFLP regimen), and high-frequency, high-pressure, and low-intensity BFR training (the HFHP regimen), while the CG only performed low-intensity training without additional pressurized training; (d) outcome indices: one-repetition maximal strength (1RM), muscle cross-sectional area (CSA), isometric muscle strength (IMS), heart rate (HR), systolic blood pressure (SBP), diastolic blood pressure (DBP).

The exclusion criteria were as follows: 1) conference papers; 2) studies with unavailable full text or incomplete data; 3) duplicate publications;4) studies without a clear description of training intensity; 5) studies using intensity indicators that cannot be converted into standard metrics (such as percentage of 1RM, percentage of maximum heart rate, or ratings of perceived exertion).

### 2.2 Retrieval strategies

Searches were conducted separately by two researchers. PubMed, Web of Science, Embase, the Cochrane Library, Scopus, EBSCOhost, CNKI, Proquest, and medRxiv were searched up to March 2025. The search terms included “BFR training,” “Elder,” and “randomized controlled trial.” Specific search strategies are illustrated in Supplementary Table S1.

### 2.3 Literature selection and data extraction

Two researchers screened the retrieved literature by reviewing titles and abstracts. Based on inclusion and exclusion criteria, the eligible studies were selected after the full-text review. Any disagreements were addressed by a third reviewer. Data were extracted using an Excel sheet, including the authors, year, sample size, age, intervention program (frequency, intensity, period), and outcome indicators.

### 2.4 Risk of bias evaluation

The risk of bias was assessed by two researchers using the Physiotherapy Evidence Database (PEDro) scale, and any disagreements were addressed by a third reviewer. 11 items were scored by the PEDro scale. Since the evaluation of item 1 was excluded, the total score was 10. When the score was ≥6, the study was considered to have a low risk of bias.

### 2.5 Statistical methods

Data preprocessing and analysis were performed independently by two researchers. The extracted data were pre-processed using Excel 2016 and converted into mean differences or standard deviations between pre- and post-intervention values. The network meta-analysis was conducted using Stata 17.0. For outcomes with consistent units, weighted mean difference (WMD) were used; for outcomes with different units, standardized mean difference (SMD) were used, along with 95% confidence intervals (CI). A network evidence diagram was plotted for direct comparisons among different combinations of intervention intensity and frequency. In this diagram, the size of each node represented the sample size, while the thickness of the connecting lines reflected the amount of direct evidence between interventions. Two methods were employed to assess inconsistency: 1) loop inconsistency testing, where a 95% CI of 0 for the inconsistency factor indicated good consistency; 2) the node-splitting method, which separated each node (intervention) into direct and indirect comparisons to evaluate the differences between them. When a significant difference indicated inconsistency, an inconsistency model was applied. When no significant difference was suggested in consistency, a consistency model was used. The optimal combination of intervention intensity and frequency was identified by the cumulative ranking probability curve (SUCRA). To evaluate the robustness of the results, sensitivity analysis was conducted by sequentially excluding individual studies and re-analyzing the data to assess the stability of the findings. Additionally, adjusted funnel plots were utilized to evaluate potential small-sample effects or publication bias. To further examine the impact of publication bias on the results, Egger’s regression test was employed to assess the asymmetry of the funnel plots.

## 3 Result

### 3.1 Literature search results

**FIGURE 1:**
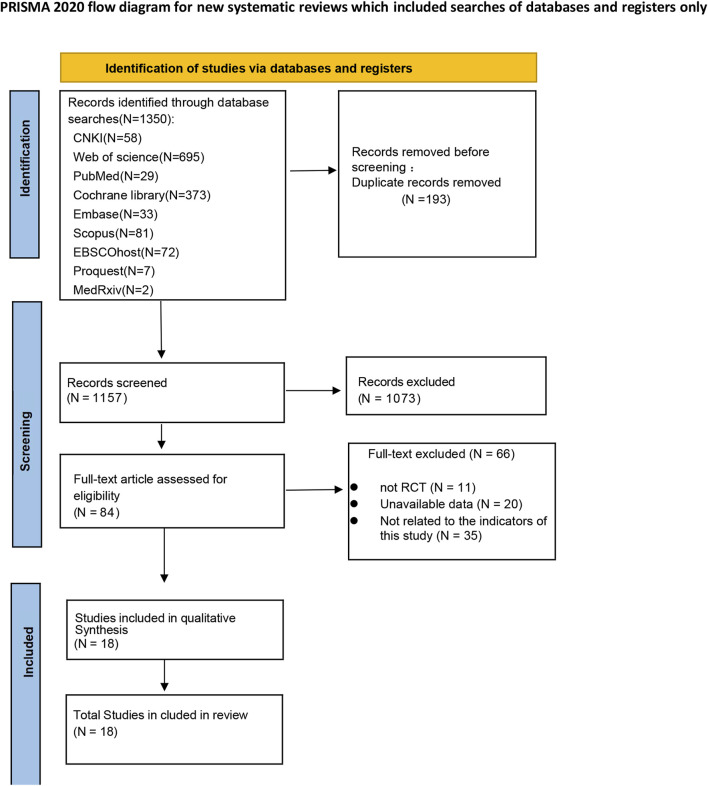
Literature retrieval flowchart.

A total of 1350 studies were selected. 84 studies remained after the initial screening. Ultimately, 18 studies were included in this net meta-analysis after a full-text review. The literature screening process is shown in Figure 1.

### 3.2 Basic characteristics of included studies

A total of 18 studies involving 626 participants, all published in English, were included. In the intervention group, two studies used LFLP interventions (Ferraz et al., 2018; Scott et al., 2018); six studies employed LFHP interventions (Vieira et al., 2013; Yasuda et al., 2013; Yasuda et al., 2014; Yasuda et al., 2015; Cook et al., 2017; Parkington et al., 2022); four studies applied HFLP interventions (Patterson and Ferguson, 2011; Letieri et al., 2018; Lopes et al., 2022); and eight studies employed HFHP interventions (Abe et al., 2010; Ozaki et al., 2010; Ozaki et al., 2011; Segal N. et al., 2015; Segal N. A. et al., 2015; Shimizu et al., 2016; Bigdeli et al., 2020). The muscle strength indices included 1RM (n = 4), CSA (n = 3), IMS (n = 3). These muscle strength indices covered various muscle groups. The cardiovascular indices included HR (n = 3), SBP (n = 4), and DBP (n = 4). The basic characteristics of the included studies are shown in Table 1.

**TABLE 1:** Basic characteristics of the included studies.

References	n	Age		Exercise protocol	The method of blood flow restriction		Measurement
		T	C		Duration/frequency	Cuff pressure (mmHg)	
Scott et al. (2018)	15women	66.8 ± 3.8	66.8 ± 3.8	leg press/leg extension LI-BFR:10 rep (20% 1RM)x3 CON: (20% 1RM)x3	2 weeks 1 day/week	Total occlusion pressure 50%	SBP DBP
Yasuda et al. (2013)	5men 14women	71.3 ± 7.1	67.7 ± 6.0	knee extension and leg press LI-BFR:75 rep (30, 20, 15, 10 reps)20%–30%1RM CON: Daily exercise	12 weeks 2 days/week	196 ± 18	1Rm CSA SBP DBP HR
Shimizu et al. (2016)	33men 7women	72 ± 4	70 ± 4	leg extension, leg press, rowing, and chest press LI-BFR: (3 × 20rep)20% 1RM CON: (3 × 20rep)20% 1RM	4 weeks 3 days/week	200	1RM SBP DBP HR
Lopes et al. (2022)	22women 10men	72 ± 7	72 ± 7	elbow flexion, leg press, pulley elbow extension, and knee extension. LI-BFR: (3 × 10rep)30% of 1 RM CON: (3 × 10rep)30% of 1 RM	12 weeks 3 days/week	65 ± 5	IMS
Patterson and Ferguson (2011)	10men	67 ± 3	67 ± 3	plantar-flexion LI-BFR: (3 × 10rep)25% 1RM CON: (3 × 10rep)25% 1RM	4 weeks 3 days/week	110	IMS 1RM
Yasuda et al. (2015)	3men 14women	72 ± 6	68 ± 5	bilateral arm curl and triceps press-down exercise training LI-BFR: (1 × 30+3 × 15rep)25% 1RM CONT: (1 × 30+3 × 15rep)25% 1RM	12 weeks 2 days/week	196 ± 18	SBP DBP HR
Yasuda et al. (2014)	5men 11women	70 ± 6	67 ± 7	knee extension and leg press LI-BFR: (20%–30%) 1RM CON:Daily exercise	12 weeks 2 days/week	120–270	CSA 1RM
Letieri et al. (2018)	56women	68.8 ± 5.0	68.8 ± 5.0	Squat/Leg Press/Knee Extension/Leg Curl LI-BFR-H: (15 repx3-4)20%–30%1RM LI-BFR-L: (15 rep x3-4)20%–30%1RM CON: 20%–30%1RM *3–4	16 weeks 3 days/week	LI + BFR-H: 185.75 ± 5.45 LI + BFR-L: 105.45 ± 6.5	IMS
Cook et al. (2017)	36men	(72.3–80.7)	(69.6–79.9)	leg extension leg curl leg press LI-BFR:3 × 15rep (30% 1-RM) CON: upper-extremity static flexibility light resistance training programme	12 weeks 2 days/week	184 ± 25	CSA 1RM
Segal et al. (2015a)	44men	56.1 ± 5.9	54.6 ± 6.9	leg press LI-BFR-: (1 × 30reps+3 × 15reps)30% 1RM. CON: (1 × 30reps+3 × 15reps)30%1RM	4 weeks 3 days/week	160–200	1RM
Vieira et al. (2013)	12men	66 ± 7	66 ± 7	single-arm biceps curl LI-BFR: (30% of 1 RM) for 3 min. CON: (30% of 1 RM)for 3 min	1 week 2 days/week	120	HR SBP DBP
Parkington et al. (2022)	10men 10women	64.3 ± 4.2	64.3 ± 4.2	leg press then knee extension LI-BFR: (1x30rep+3 × 15rep) LP 20%1RM (4 × 15rep)LE20%1RM CON: (1 × 30rep+3 × 15rep)LP20%1RM (4 × 15rep)LE20%1RM	2 weeks 1 day/week	188.3 ± 24.8	SBP DBP
Ferraz et al. (2018)	48women	60.3 ± 3	60.7 ± 4	leg press and knee extension LI-BFR: (4x15rep)30% 1RM BFRT: (4 × 15)30% 1RM	12 weeks 2 days/week	97.4 ± 7.6	1RM CSA
Segal et al. (2015b)	45women	56.1 ± 5.9	54.6 ± 6.9	leg-press resistance training. LI-BFR: (1 × 30rep+3 × 15rep)30% 1RM CON: (1 × 30rep+3 × 15rep)30% 1RM	4 weeks 3 days/week	160–200	IMS 1RM
Bigdeli et al. (2020)	30men	67.6 ± 5.1	66.3 ± 4.6	circuit training: eleven stations LI-BFR: (2–4x10rep) 25%–35% 1RM CON: (2–4x10rep) 25%–35% 1RM	6 weeks 3 days/week	126–150	1RM
Ozaki et al. (2011)	18men	64 ± 1	68 ± 1	20-min treadmill walking at 45% of heart rate reserve intensity. LI-BFR: 4.5 ± 0.0 km/h and 1.6 ± 0.4 degrees CON: 4.4 ± 0.1 km/h and 1.5 ± 0.5°	10 weeks 4 days/week	140–200	CSA IMS
Abe et al. (2010)	19 men	60∼ 78	60∼ 78	20 min treadmill walking at 45% of heart rate reserve intensity LI-BFR:67 m/min for 20 min CON: 67 m/min for 20 min	6 weeks 5 days/week	160–200	CSA IMS HR SBP DBP
Ozaki et al. (2011)	23 men and women	66 ± 1	68 ± 1	20-min treadmill walking at 45% of heart rate reserve intensity. LI-BFR: treadmill walking CON: treadmill walking exercise intensity	10 weeks 4 days/week	140–200	CSA IMS

REP: number of repetitions; LI-BFR: blood flow restriction combined with low-intensity training; LI: low-intensity training; CON: control group; LI + BFR-H: high-pressure blood flow restriction combined with low-intensity training; LI + BFR-L, low-pressure blood flow restriction training combined with low-intensity training.

### 3.3 Risk of bias evaluation results

The PEDro scores of the included studies ranged from 4 to 10, with a median score of 7. Out of the 18 studies, 12 met the predetermined threshold (≥6 points). The included studies were randomized controlled trials and clearly defined their inclusion criteria. Six studies provided detailed descriptions of allocation concealment; 13 studies reported comparable baseline characteristics; two studies implemented blinding for participants; none blinded the investigators; three studies blinded outcome assessors; 15 studies had a dropout rate exceeding 15%; and one study did not perform an intention-to-treat analysis. All studies reported between-group statistics, point estimates, and differences (as shown in Table 2).

**TABLE 2:** PEDro scale scores.

Author	Year	A	B	C	D	E	F	G	H	I	J	K	Total score
Amorim	2022	1	1	1	0	1	0	0	1	1	1	1	7
Yasuda (1)	2013	1	1	0	1	0	0	0	1	1	1	1	6
Yasuda (2)	2014	1	1	0	1	0	0	0	1	1	1	1	6
Yasuda (3)	2015	1	0	0	1	0	0	0	1	1	1	1	5
Shimizu	2016	1	1	0	1	0	0	0	1	1	1	1	6
lopes	2022	1	1	0	0	0	0	0	1	1	1	1	5
Patterson	2011	1	0	0	1	0	0	0	1	1	1	1	5
Letieri	2018	1	1	1	1	1	0	1	1	1	1	1	9
cook	2007	1	1	1	1	0	0	0	1	1	1	1	7
Segal (1)	2015	1	1	1	1	0	0	0	1	1	1	1	7
Segal (2)	2015	1	1	1	1	1	0	1	1	1	1	1	9
Vieira	2013	1	1	0	0	0	0	0	1	1	1	1	5
Parkington	2022	1	1	0	1	0	0	0	1	1	1	1	6
Ferraz	2018	1	1	1	1	0	0	0	0	1	1	1	6
Bigdeli	2020	1	1	0	0	0	0	1	0	1	1	1	5
Abe	2011	1	1	0	1	0	0	0	1	1	1	1	6
Ozaki (1)	2011	1	0	0	1	0	0	0	1	1	1	1	5
Ozaki (2)	2011	1	1	0	0	0	0	0	0	1	1	1	4

(A) Eligibility criteria specified; (B) Random allocation of subjects to groups; (C) Concealed allocation; (D) Baseline similarity; (E) Blinding of subjects; (F) Blinding of therapists; (G) Blinding of assessors; (H) >85% follow-up for key outcomes; (I) Intention-to-treat analysis; (J) Between-group statistical comparisons reported; (K) Point estimates and variability measures.

### 3.4 Net meta-analysis

#### 3.4.1 Network evidence diagram

**FIGURE 2:**
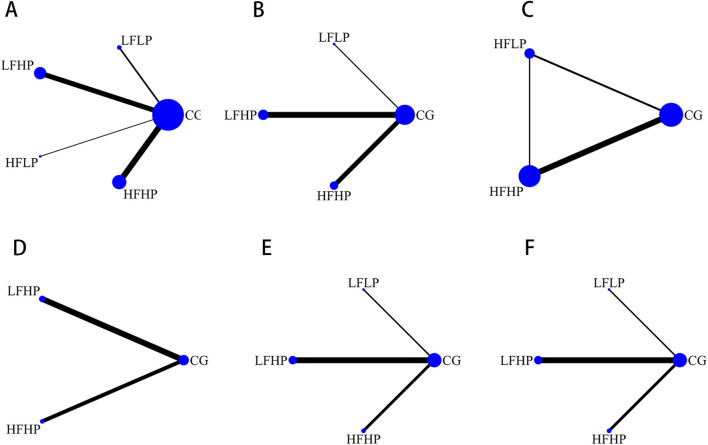
Network evidence diagram. **(A)**: 1RM **(B)**: CSA **(C)**: IMS **(D)**: HR **(E)**: SBP **(F)**: DBP.

The reticulation graphs showed that the LFHP regimen, HFHP regimen, and controls had larger sample sizes, while the LFLP regimen and the HFLP regimen had smaller sample sizes. Only the LFHP regimen, HFHP regimen, and the controls included the HR metrics. Direct comparisons between other BFR-LI intervention programs were lacking (as shown in Figure 2).

#### 3.4.2 Inconsistency analysis

**FIGURE 3:**
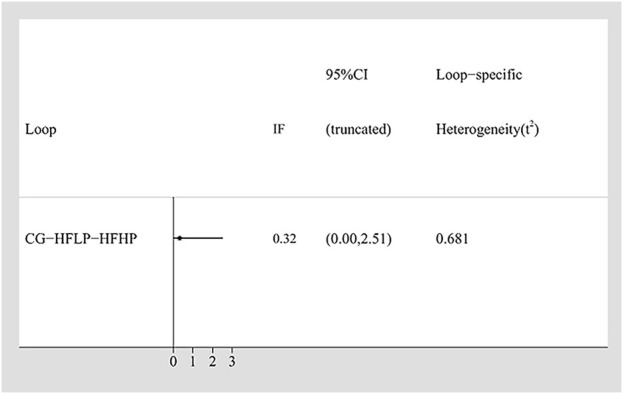
Loop consistency testing of IMS.

To assess the consistency in our network meta-analysis, the Inconsistency Factor for each closed loop was calculated. For the IMS, the Inconsistency Factor was 0.32 (95% CI: 0–2.51), with the 95% confidence interval including 0, indicating no significant inconsistency between direct and indirect comparisons (Figure 3). This result was further validated using the node-splitting method. It yielded a p-value >0.05 for IMS (Supplementary Table S2), thus confirming the good local consistency. Based on these findings, a consistency model was applied, as both the IF 95% CI included 0 and the node-splitting p-value >0.05 (Tu et al., 2014).

#### 3.4.3 Meta-analysis results

**FIGURE 4:**
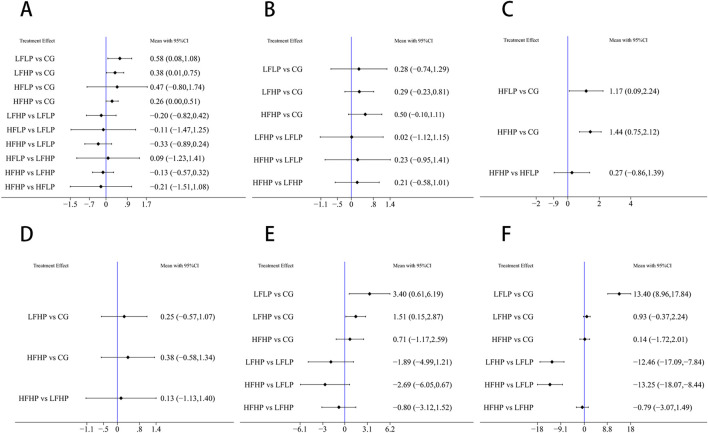
Results of reticulated meta-analysis **(A)**: 1RM **(B)**: CSA **(C)**: IMS **(D)**: HR **(E)**: SBP **(F)**: DBP.

For the 1RM outcome, compared to the CG, the LFLP intervention significantly improved 1RM (WMD = 0.58, 95% CI: 0.81–1.08, *P* < 0.05). The LFHP intervention also significantly increased 1RM (WMD = 0.38, 95% CI: 0.01–0.75, *P* < 0.05). Moreover, LFLP intervention outperformed other BFR-LI training regimens, though the difference was not statistically significant. These results indicated that the low-frequency BFR-LI regimen, whether high or low pressure, can improve 1RM (Figure 4A).

For the CSA outcome, the HFHP intervention showed greater improvement than the CG (WMD = 0.50, 95% CI: −0.10–1.11, *P* > 0.05), and HFHP intervention also outperformed other BFR-LI training regimens, though differences were not statistically significant. This suggested that HFHP intervention may be the most effective for improving CSA (Figure 4B).

For the IMS outcome, pairwise comparisons showed that HFHP intervention significantly improved IMS compared to the CG (WMD = 1.44, 95% CI: 0.75–2.12, *P* < 0.05). Similarly, HFLP intervention also significantly improved IMS (WMD = 1.17, 95% CI: 0.09–2.24, *P* < 0.05). Thus, HFHP intervention can serve as the most effective for enhancing IMS (Figure 4C).

Pairwise comparisons across 1RM, CSA, and IMS indicated that HFHP intervention was more effective in boosting muscle strength than other BFR-LI training regimens with different pressures and frequencies.

For HR, although LFHP and HFHP interventions greatly increased HR compared to controls, the differences were not statistically significant (*P* > 0.05) (Figure 4D).

For SBP, the LFLP intervention significantly increased SBP compared to controls (WMD = 3.40, 95% CI: 0.61–6.19, *P* < 0.05). LFHP intervention also significantly increased SBP compared to controls (WMD = 1.51, 95% CI: 0.15–2.87, *P* < 0.05). Other pairwise comparisons showed no significant differences (Figure 4E).

For DBP, the LFLP regimen significantly increased DBP compared to controls (WMD = 13.40, 95% CI: 8.96–17.84, *P* < 0.05). In addition, the LFLP regimen increased more DBP compared to both LFHP (WMD = −12.46, 95% CI: −17.09 to −7.84, *P* < 0.05) and HFHP regimens (WMD = −13.25, 95% CI: −18.07 to −8.44, *P* < 0.05). Other comparisons were not statistically significant (Figure 4F).

#### 3.4.4 Cumulative probability ranking results

For 1RM, CSA, and IMS outcomes, a higher cumulative area under the SUCRA curve indicated that the intervention was more effective in enhancing muscle strength in older adults. Conversely, for HR, SBP, and DBP, a higher SUCRA value suggested a more detrimental effect on cardiovascular health.

**FIGURE 5:**
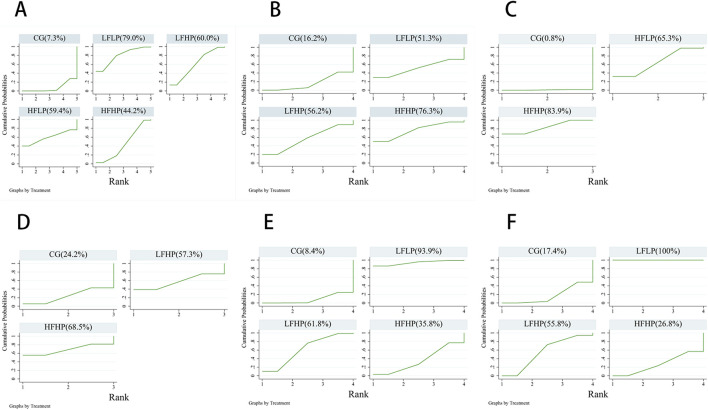
Cumulative ranked probability plots of the effects of each intervention programme on muscle strength and cardiovascular effects. **(A)**: 1RM **(B)**: CSA **(C)**: IMS **(D)**: HR **(E)**: SBP **(F)**: DBP.

The results indicated that among muscle strength measures, HFHP intervention showed the best performance for both CSA and IMS, while LFLP intervention performed best for 1RM. In cardiovascular measures, the LFLP intervention caused the greatest increases in both SBP and DBP, while the HFHP intervention led to the highest increase in HR. These findings suggested that high-pressure training performed more than three times per week may be optimal for improving muscle strength in older adults. However, older adults should limit LPLF training to maintain cardiovascular health. (Figure 5).

#### 3.4.5 Publication bias test

**FIGURE 6:**
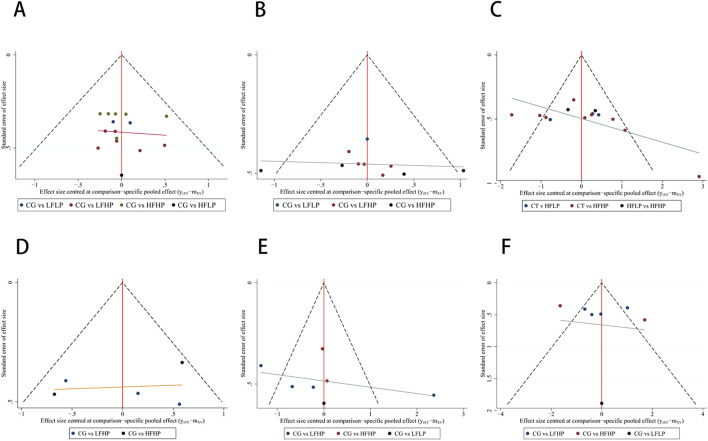
Publication bias testing. **(A)**:1RM **(B)**: CSA **(C)**: IMS **(D)**:HR **(E)**: SBP **(F)**: DBP.

The funnel plot for each indicator was symmetrical. Most of the points were evenly distributed in the funnel plot, with only a small number of points falling on the outside of it. It suggested that the results were generally even. Since publication bias may exist, the results should be interpreted with caution (Figure 6). In the assessment of publication bias, we evaluated the results using Egger’s test. Egger’s test indicated no significant publication bias for metrics such as 1RM, CSA, IMS, HR, and SBP (*p* > 0.05). However, for DBP, Egger’s test showed significant publication bias (*p* < 0.05). Given the small sample size for DBP (n < 10), the reliability of Egger’s test results is limited, and we did not proceed with the trim-and-fill method. We recommend future studies with larger sample sizes further to validate the impact of publication bias on DBP. (Supplementary Figure S1).

#### 3.4.6 Subgroup analysis

This study conducted subgroup analyses based on gender. The participants were divided into male, female, and mixed groups to analyse the effects of different pressurization training regimens on 1RM.

In the female group, the LFLP regimen showed statistical significance compared to the control group [WMD = 0.58, 95% CI (0.08, 1.08), *P* < 0.05] (Supplementary Figure S2B). The results indicated that the LFLP regimen ranked highest in the cumulative probability ranking for 1RM values (Supplementary Figure S2C). The funnel plots for the indicators were largely symmetrical, with most points evenly distributed (Supplementary Figure S2D).

In the male group, the HFHP regimen indicated statistical significance compared to the control group [WMD = 0.46, 95% CI (0.06, 0.86), *P* < 0.05] (Supplementary Figure S3B). The results suggested that the HFHP intervention ranked highest in the cumulative probability ranking for 1RM values (Supplementary Figure S3C). The funnel plots for the indicators were largely symmetrical, with most points evenly distributed (Supplementary Figure S3D).

For the mixed group, pairwise comparisons revealed no significant differences among the various regimens. No statistical significance was observed when comparing each BFR regimen with the control group (Supplementary Figure S4B). The results indicated that the LFLP intervention ranked highest in the cumulative probability ranking for 1RM values (Supplementary Figure S4C). The funnel plots for the indicators were largely symmetrical, with most points evenly distributed (Supplementary Figure S4D).

#### 3.4.7 Sensitivity analysis

Since the age of the included participants mainly ranged between 60 and 70 years, a sensitivity analysis was conducted by excluding studies with higher age ranges. The results showed that the differences in the outcomes for the effects of different BFR-LI training on 1RM (Supplementary Figure S5) and IMS (Supplementary Figure S6) among older adults were minimal before and after the exclusion. Therefore, the results of this study are relatively reliable and stable.

## 4 Discussion

This net meta-analysis aims to investigate the effects of various BFR-LI training regimens combined with low-intensity exercise on muscle strength and cardiovascular function in older adults. We integrated four exercise regimens based on different pressure levels (high-pressure BFR ≥120 mmHg; low-pressure BFR <100 mmHg) and exercise frequencies (low frequency <3 sessions per week; high frequency ≥3 sessions per week) to identify the optimal BFR-LI training strategy. This net meta-analysis revealed that under high-pressure conditions, BFR-LI exercise performed three or more times per week yielded greater improvements in both CSA and IMS. Conversely, under low-pressure conditions, BFR-LI exercise performed less than three times per week resulted in better improvements in 1RM. However, under low-pressure conditions, there was a significant increase in both systolic and diastolic blood pressures in the BFR-LI training, while HR remained unaffected by changes in pressure or frequency.

Regarding muscle strength outcomes, both the chosen pressure level and training frequency significantly influenced post-training muscle growth in older adults. Our analysis demonstrated that the HFHP regimen was the most effective for increasing muscle strength. Specifically, high-pressure conditions, on average, were more effective in enhancing muscle strength than low-pressure conditions. This finding is consistent with previous studies which suggested that under high cuff pressures (mean > 120 mmHg), low-intensity exercise at high pressures can yield muscle strength improvements comparable to those achieved with high-intensity exercise at high pressures (Mahmoud et al., 2021). When using very low exercise loads, higher BFR pressures may be more conducive to muscle growth (Dankel et al., 2017). The underlying reasons for these effects are twofold. First, from a hemodynamic perspective, high-pressure BFR-LI leads to greater hemodynamic responses, including significant increases in HR, blood pressure, cardiac output, and the rate-pressure product, compared to low-pressure BFR-LI (Mouser et al., 2019). Second, there are differences in muscle activation. High-pressure BFR-LI may enhance muscle activation, whereas low-pressure BFR-LI might reduce it (Hwang and Willoughby, 2019). This difference potentially influences the degree of muscle strength and hypertrophy achieved (Brandner et al., 2015). Under high-pressure conditions, reduced blood flow to active muscles can result in the accumulation of inorganic phosphate, which induces muscle fatigue (Lixandrão et al., 2015) and prompts the recruitment of fast-twitch fibers and additional motor units to preserve force production, ultimately leading to muscle hypertrophy (Schoenfeld et al., 2019). Furthermore, several studies have shown that low-intensity exercise under low-pressure BFR-LI also contributes to muscle strength gains. For example, when performing elbow flexion exercises at 40% and 90% of arterial occlusion pressure in older adults, no significant differences in strength gains were observed between different pressure levels (Counts et al., 2016). This suggests that lower BFR-LI pressures can be effectively used during training. Regarding training frequency, our analysis indicated that high-pressure training required at least three sessions per week to optimally enhance muscle strength. Increased training frequency enhances muscular adaptations by promoting greater muscle fiber recruitment, activating metabolic pathways, and stimulating protein synthesis (Schoenfeld et al., 2019). Under high-pressure BFR-LI, higher training frequencies can elevate metabolic stress, including the accumulation of lactate and other metabolites, which is a key factor in promoting muscle growth (Moesgaard et al., 2022). Although some studies have suggested that as long as the total training volume is sufficient, lower frequencies can also induce muscle hypertrophy (Neves et al., 2022), the relatively low exercise load in BFR-LI exercise makes increasing frequency a more effective strategy to promote muscle activation. Studies have found that there are differences in the responses to pressurization training between genders. In the female group, the LFLP regimen significantly improved 1RM, while in the male group, the HFHP regimen was more effective in improving 1RM. This suggests that men and women may have different requirements for the intensity and frequency of pressurization training in muscle strength exercise. This difference may potentially be due to variations in physiological structure, hormone levels, or types of muscle fiber. In the subgroup analyses, the funnel plots were largely symmetrical with evenly distributed points, indicating minimal bias in the study results and high reliability of the data. This supports the credibility of the study’s conclusions and indicates the rigor of the study design and methodology.

This network meta-analysis revealed that a LFLP regimen was associated with more adverse cardiovascular effects. Regarding pressure, cuff pressure is one of the most important factors influencing the safety of BFR-LI training. Our findings are controversial compared to previous studies, which have indicated that following the LFLP regimen, the cardiovascular load is lower than that observed with high-intensity exercise without BFR (Ferreira et al., 2017). However, our study further observed that under low-pressure BFR-LI conditions, the cardiovascular responses during low-intensity exercise were more pronounced. Specifically, during low-pressure BFR-LI exercise, HR, SBP, and DBP increased significantly compared to both pure low-intensity exercise and other BFR exercises. This phenomenon may be related to exercise frequency. BFR-LI training has a dual impact on the autonomic nervous system. In the short term, BFR-LI training may activate the sympathetic nervous system and increase heart rate and blood pressure by partially restricting blood flow, thus leading to local muscle hypoxia and the accumulation of metabolites (such as lactate and hydrogen ions). These metabolites send signals to the central nervous system by stimulating type III and IV afferent fibers in the muscles, thus activating the sympathetic nervous system and increasing heart rate and blood pressure to cope with local hypoxia and metabolic stress, especially during high-pressure training (Igor S, 2021). In the long term, BFR-LI training may increase the release of nitric oxide (NO) and promote vasodilation by enhancing the activity of the parasympathetic nervous (Ferreira Junior et al., 2019) and improving vascular function (Shimizu et al., 2013; Killinger et al., 2020). The enhancement of parasympathetic nervous activity helps balance the overactivation of the sympathetic nervous system, thereby improving heart rate variability and blood pressure regulation, and reducing cardiovascular risk (Ma et al., 2024). The included studies that investigated low-pressure BFR-LI training involved relatively low exercise frequencies and durations and did not cover the impact of the HFLP regimen on cardiovascular outcomes. Therefore, future studies should explore the HFLP regimen to comprehensively evaluate the effects of different frequencies on cardiovascular health in older adults. Some studies have pointed out that due to excessive cuff pressure, high-pressure BFR-LI training can induce vascular occlusion that may trigger excessive muscle reflex activation (such as the activation of type III and IV afferent fibers), thus resulting in overactivation of the sympathetic nervous system, increased HR, elevated blood pressure, and increased vascular resistance (Sacino and Rosenblatt, 2019). However, the HFHP regimen can produce adaptive responses in older adults. Long-term BFR-LI training may reduce the muscle’s sensitivity to accumulated metabolites and signaling molecules, thereby potentially mitigating the training-induced increases in systolic blood pressure (Cho and Lee, 2024) and resulting in less cardiovascular harm. In contrast, LFLP training may provoke a marked acute increase in HR, systolic, and diastolic blood pressures. These responses are similar to those seen in high-intensity exercise (Zhang et al., 2022), which could elevate the risk of cardiovascular adverse events and neural injury (Sacino and Rosenblatt, 2019), causing greater discomfort for participants. This discrepancy may be attributed to age-related changes in the autonomic nervous system and neural aging in individuals who lack long-term regular physical exercise, which affects blood pressure regulation (Liu et al., 2022), thereby significantly increasing the risk of cardiovascular events during and after exercise.

The impact of BFR-LI training on cardiovascular health in older adults is complex. For individuals who cannot tolerate high mechanical stress on joints and suffer from skeletal muscle dysfunction, BFR-LI trainig may offer benefits (Rolnick et al., 2022). However, in patients with chronic conditions such as hypertension (Bezerra Silveira et al., 2022), diabetes (Camm et al., 2018), and chronic inflammation, improper use of BFR may increase the risk of cardiovascular diseases.

In terms of venous thrombosis, the effects of BFR-LI training on older adults are dual-sided: on one hand, it improves blood circulation by increasing the shear stress of blood flow, enhancing endothelial function, and promoting collateral circulation (Pereira-Neto et al., 2021). BFR-LI training also facilitates fibrinolysis by increasing tissue plasminogen activator (t-PA), reducing plasminogen activator inhibitor-1 (PAI-1), and activating the fibrinolytic system, thereby lowering the risk of thrombosis (Landers et al., 2025); on the other hand, it may increase the risk of thrombosis due to blood stasis, endothelial injury, and hypercoagulability (Engbers et al., 2010). Blood stasis occurs because older adults often have slower blood flow (Rosendaal et al., 2007), and BFR-LI training may further exacerbate this stasis. Endothelial injury is due to mechanical damage to vascular endothelial cells resulting from improper use of the cuff or excessive pressure during BFR-LI training (Chulvi-Medrano et al., 2023). Additionally, BFR-LI training may activate the coagulation system and inflammatory responses, causing a hypercoagulable state. Since older adults already have stronger coagulation function, BFR-LI training may further increase the risk of thrombosis. BFR-LI training should be conducted under professional guidance, with comprehensive screening and for thrombosis risks in older adults to ensure safety and effectiveness. (Heitkamp, 2015).

In future studies, it is essential to further investigate the impact of the HFLP regimen and the HFHP- regimen on cardiovascular indices in older populations. Although existing studies have revealed that the LFLP regimen has a pronounced effect on improving 1RM, the potential for LFLP training to trigger substantial cardiovascular responses in older adults must be considered. Therefore, considering the need to enhance muscle strength while preserving cardiovascular health, the HFHP regimen may represent a more suitable training strategy. This regimen aims to optimize the parameters of BFR-LI training to effectively boost muscle strength in older individuals while minimizing potential cardiovascular risks. It is recommended to further investigate the long-term effects of BFR-LI in different populations (such as older adults, and patients with chronic diseases) and clarify its safety and indications.

## 5 Limitation

1) The daily training habits of participants varied; the BFR-LI application sites were inconsistent; and the lack of blinding may led to uncertain outcomes. 2) Heterogeneity may occur in the cardiovascular effects of different training modalities on older adults. 3) Due to the limited number of studies included, statistical significance may not have been established; however, clinical significance cannot be ruled out. 4) Considering the limited literature and the insufficient data on individual differences and long-term safety, a systematic review of future studies should be conducted to refine our findings and strengthen data support for further research.

## 6 Conclusion

The HFHP regimen (pressures above 120 mmHg, 3 days per week) effectively enhances muscle strength and maintains cardiovascular health in older adults. In contrast, the LFLP regimen, though enhancing 1RM, may not be suitable for those with cardiovascular issues due to stronger physiological responses. The cardiovascular effects of the HFLP regimen remain unstudied and warrant further investigation.

When conducting research on different BFR-LI regimens, it is also important to consider various factors. In BFR-LI training, while training frequency and pressure magnitude are critical, other factors such as band size, duration, and intervals should also be considered to optimize the regimen. Although the HFHP regimen is beneficial, the cardiovascular risks associated with BFR-LI training remain uncertain. These risks, such as blood stasis and thrombosis, are particularly concerning for high-risk individuals, including those with hypertension or diabetes.

To address these challenges, Tailoring protocols based on gender, goals, and physiological traits can maximize benefits and reduce risks. Further research is needed to enhance BFR-LI training’s safety and effectiveness across diverse populations.

## Data Availability

The original contributions presented in the study are included in the article/Supplementary Material, further inquiries can be directed to the corresponding author.
