# Supplementary figures and images for: Effect of different blood flow restriction training regimens combined with low-intensity training on muscle strength and cardiovascular safety in older adults: a systematic review and network meta-analysis
Source: Front Physiol. 2025 Apr 28;16:1587876. doi: 10.3389/fphys.2025.1587876 (PMC12066469; doi:10.3389/fphys.2025.1587876)

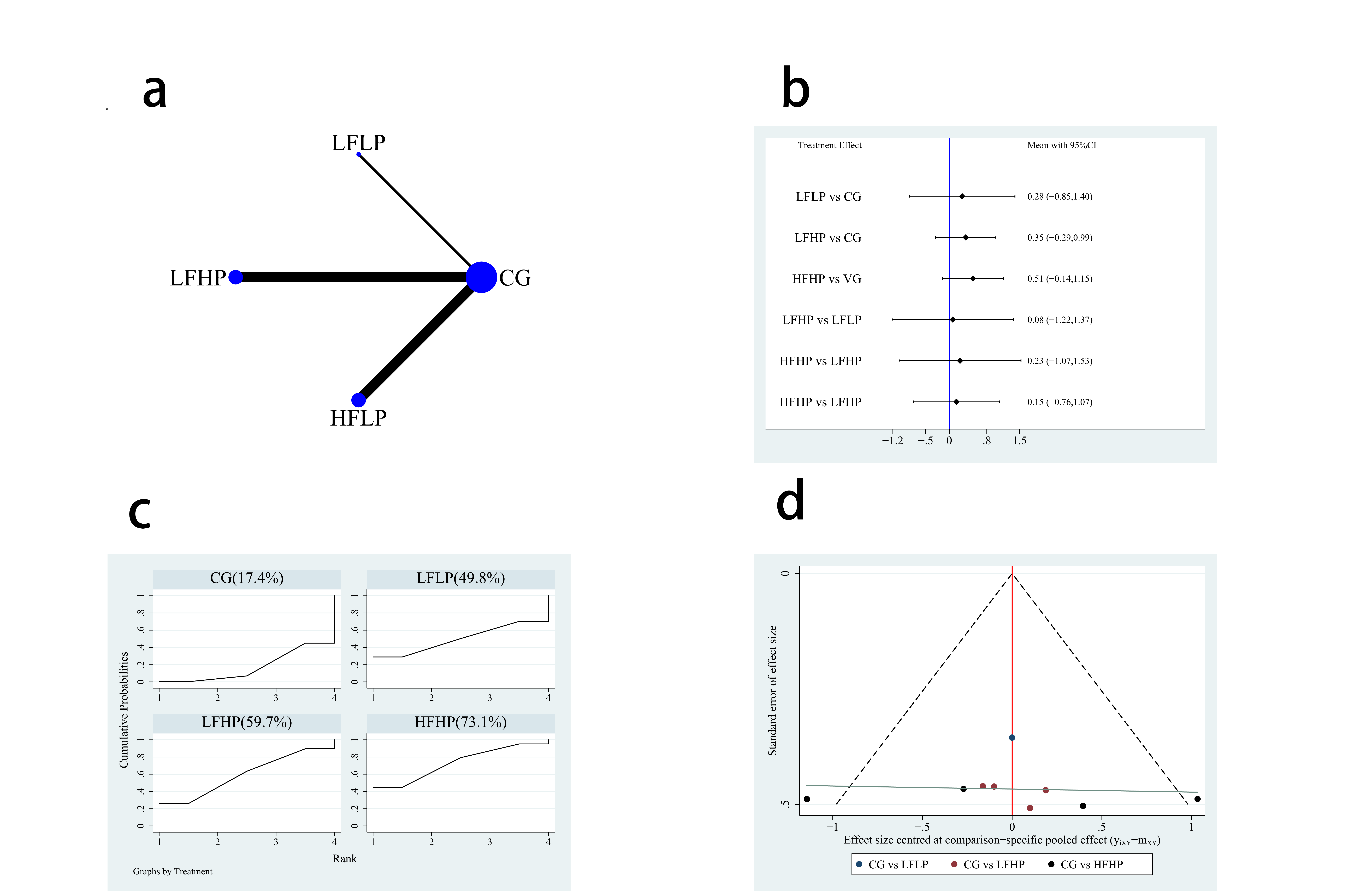

Supplement: Supplementary file 1 [file Image6.tif]

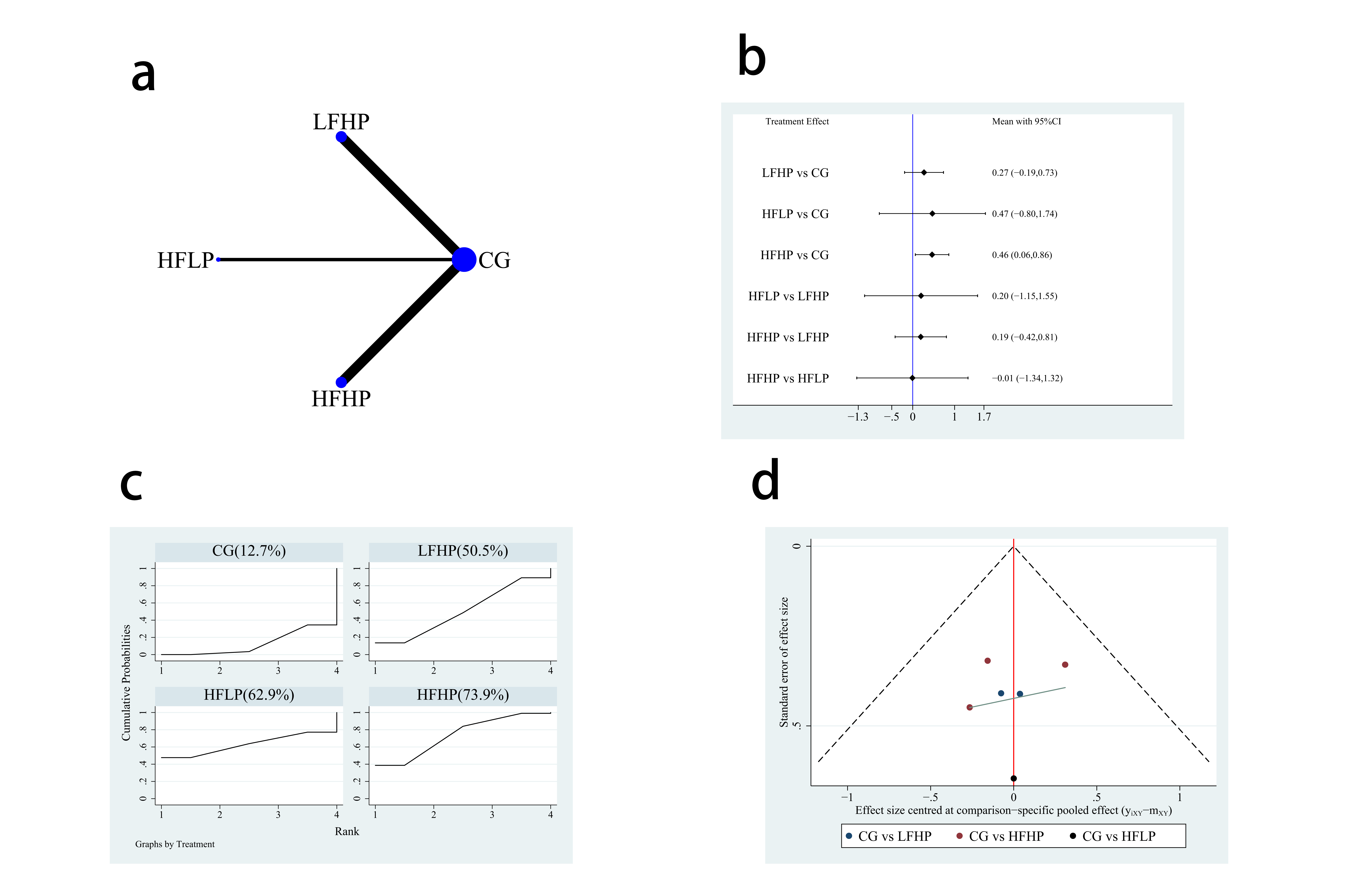

Supplement: Supplementary file 3 [file Image3.tif]

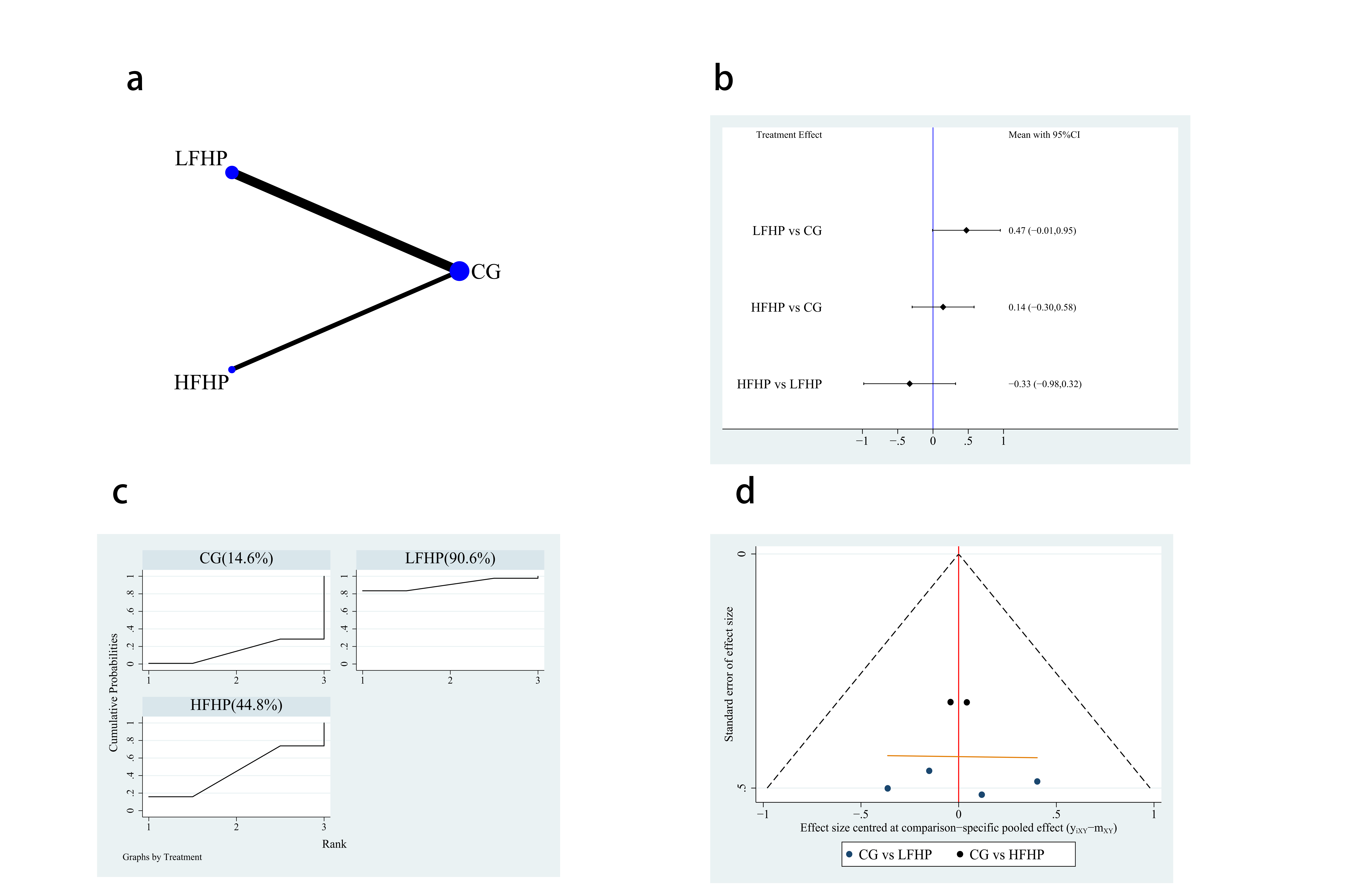

Supplement: Supplementary file 4 [file Image4.tif]

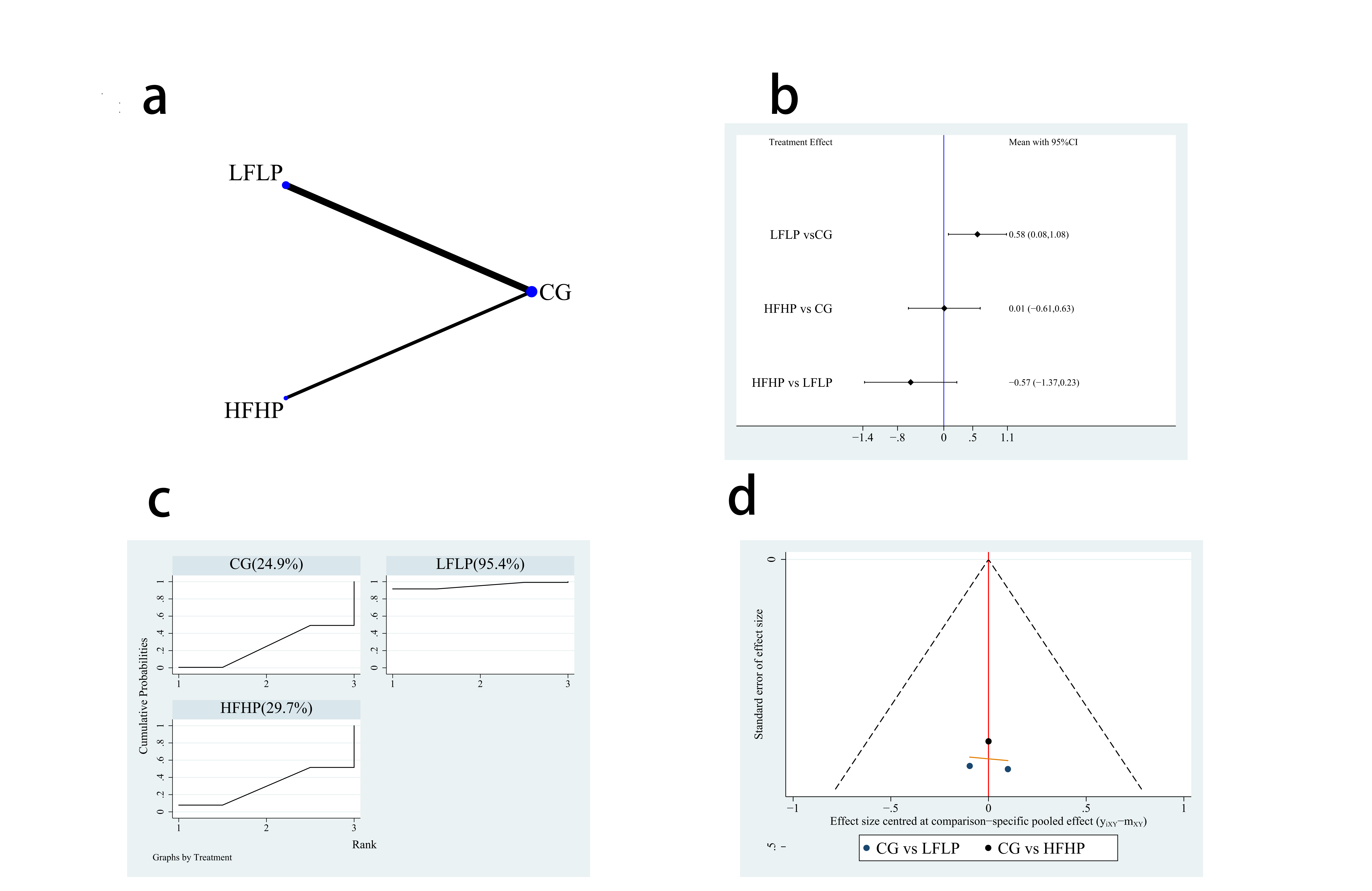

Supplement: Supplementary file 5 [file Image2.tif]

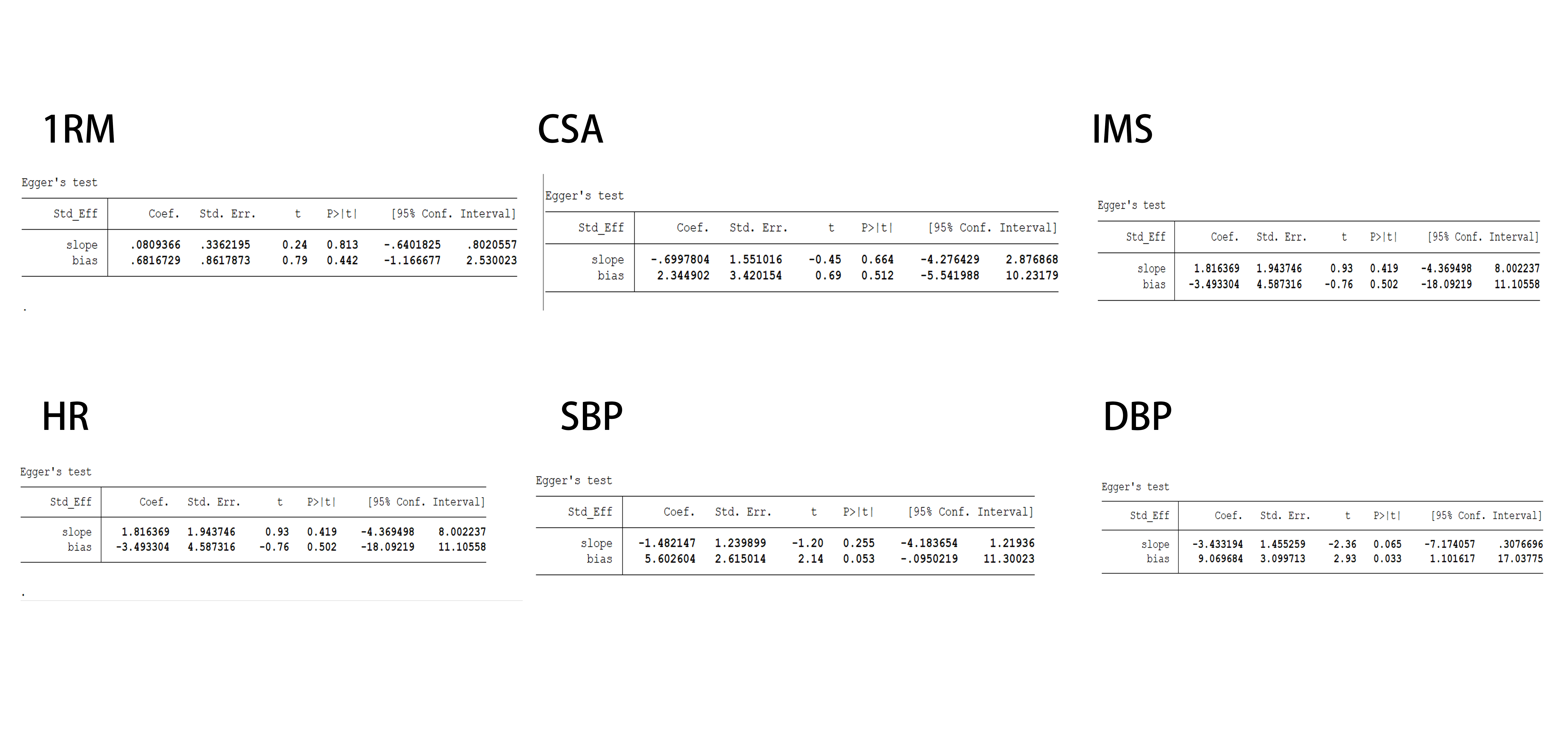

Supplement: Supplementary file 6 [file Image1.tif]

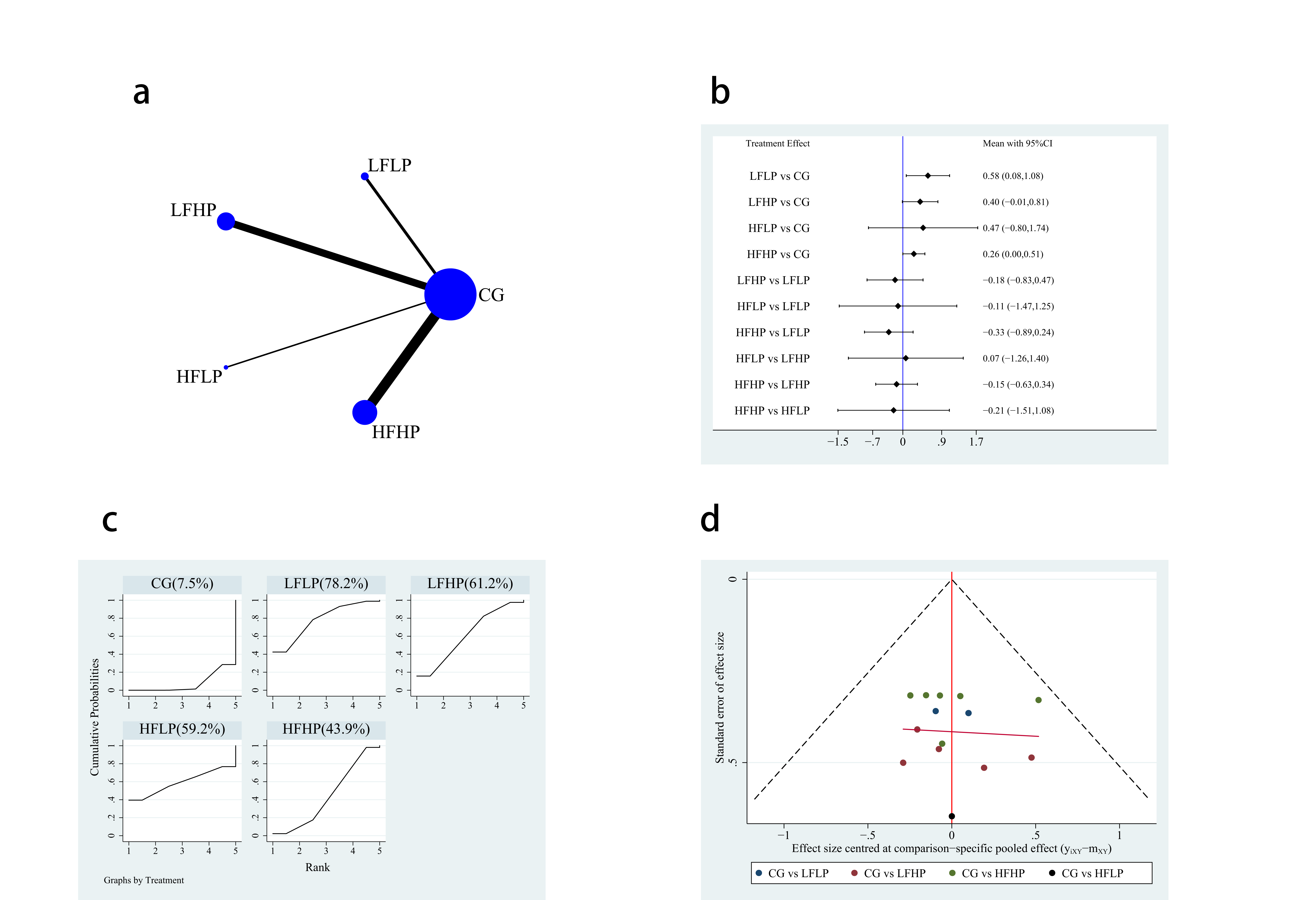

Supplement: Supplementary file 8 [file Image5.tif]
