# Supplementary material for: Effect of different blood flow restriction training regimens combined with low-intensity training on muscle strength and cardiovascular safety in older adults: a systematic review and network meta-analysis
Source: Front Physiol. 2025 Apr 28;16:1587876. doi: 10.3389/fphys.2025.1587876 (PMC12066469; doi:10.3389/fphys.2025.1587876)
Supplement: Supplementary file 2 [file Table1.docx]

| **Search strategies** | | | |
| --- | --- | --- | --- |
|  | Search number | Query | Total |
| Pubmed | #1 | (((((Elder[Title/Abstract]) OR (Elderly[Title/Abstract])) OR (older adults[Title/Abstract])) OR (frail elder[Title/Abstract])) OR (functionally impaired elderly[Title/Abstract])) OR (frail older adult[Title/Abstract]) | 420451 |
|  | #2 | (((((((((((blood flow restriction training[Title/Abstract]) OR (BFR Therapy[Title/Abstract])) OR (BFR Therapies[Title/Abstract])) OR (Blood Flow Restriction Exercise[Title/Abstract])) OR (kaatsu training[Title/Abstract])) OR (occlusion training[Title/Abstract])) ) OR (blood flow restricted[Title/Abstract])) OR (blood flow restriction[Title/Abstract])) OR (kaatsu[Title/Abstract])) OR (tourniquets[Title/Abstract])) OR (vascular occlusion[Title/Abstract]) | 7608 |
|  | #3 | (((randomized controlled trial[Title/Abstract]) OR (randomized[Title/Abstract])) OR (placebo[Title/Abstract])) OR (RCT[Title/Abstract]) | 838648 |
|  | #4 | #1 AND #2 AND #3 | 29 |
| Web of science | #1 | AB=( Elder or Elderly or older adults or frail elder or functionally impaired elderly or frail older adult) | 2716253 |
|  | #2 | AB=(blood flow restriction training or BFR Therapy or BFR Therapies or Blood Flow Restriction Exercise or kaatsu training or occlusion training or blood flow restricted or blood flow restriction or kaatsu or tourniquets or ischemia or vascular occlusion ) | 266440 |
|  | #3 | AB=( randomized controlled trial or randomized or placeboor or RCT) | 954969 |
|  | #4 | #1 AND #2 AND #3 | 695 |
| CNKI | #1 | AB=(blood flow restriction training or BFR Therapy or BFR Therapies or Blood Flow Restriction Exercise or kaatsu training or occlusion training or blood flow restricted or blood flow restriction or kaatsu or tourniquets or ischemia or vascular occlusion ) | 6349 |
|  | #2 | AB=( Elder or Elderly or older adults or frail elder or functionally impaired elderly or frail older adult) | 285800 |
|  | #3 | #1 AND #2 | 58 |
| Cochrane Library | #1 | （Elder or Elderly or older adults or frail elder or functionally impaired elderly or frail older adult）：ti,ab,kw | 129486 |
|  | #2 | （blood flow restriction training or BFR Therapy or BFR Therapies or Blood Flow Restriction Exercise or kaatsu training or occlusion training or blood flow restricted or blood flow restriction or kaatsu or tourniquets or vascular occlusion）ti,ab,kw | 9582 |
|  | #3 | （randomized controlled trial or randomized or placeboor or RCT）ti,ab,kw | 1255319 |
|  | #4 | #1 AND #2 AND #3 | 373 |
| Scopus | #1 | Elder or Elderly or older adults or frail elder or functionally impaired elderly or frail older adult | 20826 |
|  | #2 | blood flow restriction training or BFR Therapy or BFR Therapies or Blood Flow Restriction Exercise or kaatsu training or occlusion training or blood flow restricted or blood flow restriction or kaatsu or tourniquets or vascular occlusion | 1703 |
|  | #3 | randomized controlled trial or randomized or placeboor or RCT | 1071165 |
|  | #4 | #1 AND #2 AND #3 | 81 |
| Ebscohost | #1 | Elder or Elderly or older adults or frail elder or functionally impaired elderly or frail older adult | 936739 |
|  | #2 | blood flow restriction training or BFR Therapy or BFR Therapies or Blood Flow Restriction Exercise or kaatsu training or occlusion training or blood flow restricted or blood flow restriction or kaatsu or tourniquets or vascular occlusion | 43058 |
|  | #3 | randomized controlled trial or randomized or placeboor or RCT | 1310642 |
|  | #4 | #1 AND #2 AND #3 | 72 |
| Embase | #1 | ('randomized controlled trial':ab,ti OR randomized:ab,ti OR placeboor:ab,ti OR rct:ab,ti) AND ('blood flow restriction training':ab,ti OR 'bfr therapy':ab,ti OR 'bfr therapies':ab,ti OR 'blood flow restriction exercise':ab,ti OR 'kaatsu training':ab,ti OR 'occlusion training':ab,ti OR 'blood flow restricted':ab,ti OR 'blood flow restriction':ab,ti OR kaatsu:ab,ti OR tourniquets:ab,ti OR 'vascular occlusion':ab,ti) AND (elder:ab,ti OR elderly:ab,ti OR 'older adults':ab,ti OR 'frail elder':ab,ti OR 'functionally impaired elderly':ab,ti OR 'frail older adult':ab,ti) | 33 |
| Proquest | #1 | “Elder or Elderly or older adults or frail elder or functionally impaired elderly or frail older adult”AND blood flow restriction training or BFR Therapy or BFR Therapies or Blood Flow Restriction Exercise or kaatsu training or occlusion training or blood flow restricted or blood flow restriction or kaatsu or tourniquets or ischemia or vascular occlusion AND”randomized controlled trial or randomized or placeboor or RCT” | 7 |
| medRxiv | #1 | AT=”Blood Flow Restriction Exercise or kaatsu training or occlusion training or blood flow restricted or blood flow restriction or kaatsu or tourniquets or ischemia or vascular occlusion” | 2 |
|  |  |  | 1350 |
