# Supplementary material for: Effect of different blood flow restriction training regimens combined with low-intensity training on muscle strength and cardiovascular safety in older adults: a systematic review and network meta-analysis
Source: Front Physiol. 2025 Apr 28;16:1587876. doi: 10.3389/fphys.2025.1587876 (PMC12066469; doi:10.3389/fphys.2025.1587876)
Supplement: Supplementary file 7 [file Table2.docx]

| Side | Direct coef | Std.Err | Indirect Coef | Std .err | Difference coef | Std.Err | p>\|z\| | Tau |
| --- | --- | --- | --- | --- | --- | --- | --- | --- |
| CG vs LFLP | 1.303041 | 0.6168219 | 0.3754717 | 1.545728 | 0.9275689 | 1.674392 | 0.589 | 0.9561951 |
| CG vs LFHP | 1.479629 | 0.3695376 | -0.2456077 | 2.528249 | 1.725237 | 2.556585 | 0.500 | 0.9623217 |
| LFLP vs LFHP | 0.3088163 | 0.767408 | 0.2148147 | 1.045669 | 0.0940017 | 1.29925 | 0.942 | 0.9963078 |

Supplement Table 2: Node splitting method results of IMS
